# Automatic Cognitive Fatigue Detection Using Wearable fNIRS and Machine Learning

**DOI:** 10.3390/s22114010

**Published:** 2022-05-25

**Authors:** Rui Varandas, Rodrigo Lima, Sergi Bermúdez I Badia, Hugo Silva, Hugo Gamboa

**Affiliations:** 1 LIBPhys (Laboratory for Instrumentation, Biomedical Engineering and Radiation Physics), Faculdade de Ciências e Tecnologia, Universidade Nova de Lisboa, 2829-516 Caparica, Portugal; hgamboa@fct.unl.pt; 2 PLUX Wireless Biosignals S.A., 1050-059 Lisboa, Portugal; hsilva@lx.it.pt; 3 Departamento de Engenharia Informática, Universidade da Madeira & Madeira N-LINCS, 9020-105 Funchal, Portugal; rodrigo.lima@arditi.pt (R.L.); sergi.bermudez@uma.pt (S.B.I.B.); 4 NOVA Laboratory for Computer Science and Informatics, 2829-516 Caparica, Portugal; 5 Instituto de Telecomunicações (IT), 1049-001 Lisbon, Portugal; 6 Instituto Superior Técnico, Universidade de Lisboa, 1049-001 Lisbon, Portugal

**Keywords:** cognitive fatigue, functional near-infrared spectroscopy, machine learning, brain–computer interface

## Abstract

Wearable sensors have increasingly been applied in healthcare to generate data and monitor patients unobtrusively. Their application for Brain–Computer Interfaces (BCI) allows for unobtrusively monitoring one’s cognitive state over time. A particular state relevant in multiple domains is cognitive fatigue, which may impact performance and attention, among other capabilities. The monitoring of this state will be applied in real learning settings to detect and advise on effective break periods. In this study, two functional near-infrared spectroscopy (fNIRS) wearable devices were employed to build a BCI to automatically detect the state of cognitive fatigue using machine learning algorithms. An experimental procedure was developed to effectively induce cognitive fatigue that included a close-to-real digital lesson and two standard cognitive tasks: Corsi-Block task and a concentration task. Machine learning models were user-tuned to account for the individual dynamics of each participant, reaching classification accuracy scores of around 70.91 ± 13.67 %. We concluded that, although effective for some subjects, the methodology needs to be individually validated before being applied. Moreover, time on task was not a particularly determining factor for classification, i.e., to induce cognitive fatigue. Further research will include other physiological signals and human–computer interaction variables.

## 1. Introduction

Wearable physiological sensors have been used for the past years in healthcare monitoring systems and are currently seen as promising for data generation in healthcare [1,2]. The main advantages of wearable devices are that they are noninvasive, mostly non-obtrusive, less expensive than gold-standard research devices, usually comfortable to wear, and affordable to consumers [3]. Moreover, wearable systems are easily used in real-life experiments or controlled experiments with high ecological validity, allowing studies in naturalistic settings [3].

One of the areas in which wearable sensors have been applied is in the study of human psychophysiology using Brain–Computer Interfaces (BCI) [4]. BCI systems consist on the automatic communication between the brain and the computer, usually using specific sensing devices [5]. Such interfaces may be active, in which case the computer is consciously controlled by the person connected to the BCI, or passive, in which the computer monitors and possibly adapts to the mental state of the person [5]. Given these definitions, passive BCI’s can be used during learning to improve its efficacy, namely, by monitoring mental processes during learning periods. The performance of BCI systems tend to decrease with time of use, as maintaining a high performance BCI system is usually difficult due to frustration, attention, and cognitive fatigue [6].

Cognitive fatigue can be defined as the difficulty of initiating or sustaining voluntary tasks and its onset is usually triggered by performing complex mental activities over time [7]. Thus, this cognitive state negatively impacts human performance.

This work is part of a project that studies multiple aspects of learning using biosignals and human–computer interaction features. Those are explored during the execution of the presented tasks, mainly, during the digital lesson that represents a close-to-real learning scenario. The objective of the study is to advise e-learning users and to adapt the e-learning contents to ultimately optimise the learning process.

In this work, we explored the process of cognitive fatigue. This state hinders learning by impairing executive functioning, which involves the ability to update information in memory. Moreover, cognitive fatigue leads to increased perseverance, which is not beneficial in cases where subjects are unable to evaluate their state, persisting in irrational actions [7]. Thus, we developed an experimental procedure that effectively induces cognitive fatigue and monitors the participants’ mental state using a set of physiological sensors to obtain the corresponding physiological signals. The participants’ states were monitored using the electrocardiogram (ECG), electrodermal activity (EDA), and respiratory inductance plethysmography (RIP) to measure the sympathetic nervous system changes, the electroencephalogram (EEG) and functional near-infrared spectroscopy (fNIRS) to monitor cognitive processes, and an accelerometer (ACC) attached to the side of the head to measure posture changes and the overall movement of the head.

We focused our attention on the two-channel fNIRS sensor for the automatic cognitive fatigue detection, which can easily be used as a wearable sensor, providing ecological validity outside the laboratory. We applied specific signal processing techniques, extracted a set of features from the acquired signals, employed a features selection algorithm and trained a set of user-tuned machine learning (ML) models to detect the state of cognitive fatigue, i.e., the binary case of *cognitive fatigue* vs. *absence of cognitive fatigue*.

The main contributions of this paper are: (1) the development of an experimental procedure to induce cognitive fatigue; (2) study the applicability of two-channel fNIRS sensors to monitor cognitive fatigue; and (3) train and discuss how ML models can be applied in the context of cognitive fatigue detection. Thus, the hypothesis we tested was: Is it possible to automatically detect cognitive fatigue using two-channel fNIRS sensor?

This paper is divided as follows: Section 2 presents relevant related works; Section 3 describes all materials, experimental procedure, signal processing, features extraction and selection, and ML model’s evaluation description; Section 4 presents the achieved results; Section 5 and Section 6 discuss the results and presents the conclusions and future work, respectively.

## 2. Related Work

Cognitive fatigue has been extensively studied, and its assessment is usually made given one of three methods: subjective methods—the assessment is typically made via questionnaire, being subjective to each person; behavioural methods—study the relation between specific behaviours and cognitive fatigue; physiological-based methods—use physiological sensors to monitor the person and then, by processing the acquiring biosignals, relate them with cognitive fatigue [8].

Subjective methods employ questionnaires such as Situational Fatigue Scale [9], State-Trait Inventory for Cognitive Fatigue (STI-CF) [10], or Subjective Exercise Experiences Scale (SEES) [11], which can only be applied at the end of the given tasks. Thus, although useful to assess the real personal perception of cognitive fatigue, these are not applicable unobtrusively, because there needs to be an interruption to answer them. Moreover, such tests are bound in time—the answers are respective to a given instant.

Behavioural methods, however, can be employed in a time-continuous fashion. In these methods, personal behaviour is tracked using characteristics of the specific tasks at hand, meaning that it is possible to monitor cognitive fatigue over time. However, there is a cost—different tasks involve different types of behaviour, e.g., the behaviour during driving and during computer-based tasks are not comparable. Thus, these methods are specific to different tasks and can be employed in everyday life. Such methods include eye-tracking [12] and human–computer interaction features [13].

On the other hand, physiological-based methods can be continuously applicable and are agnostic to the type of activity or behaviour. In this case, wearable sensors such as EEG [14] and fNIRS [15] can be applied to monitor the mental state of subjects. Then, specific information can be drawn from the generated physiological signals to classify the mental states of the monitored individuals, including the state of cognitive fatigue. The main disadvantage is that this monitoring involves using external instruments (the sensors), which might interfere with the natural behaviour of the monitored subjects.

fNIRS sensors, in particular, have been applied because they may be used during activities of daily life, although specific processing challenges arise, namely, motion artefacts and physiological artefacts removal, specifically from heart rate and respiration rate interference, which need to be considered [16].

Cognitive fatigue assessment using fNIRS has been applied in multiple domains, such as image interpretation in medical settings [15], physical performance [17], operational settings [7], during simulated driving [14], and during serious games [13]. However, there is still a lack of evaluation of cognitive fatigue during learning.

Nihashi et al. studied the relation between the concentration of oxygenated haemoglobin with cognitive fatigue in radiologists using fNIRS sensors, where they found that, in most cases, the concentration of this chromophore was the lowest when the fatigue was the highest [15].

Analogously, Dehais et al. studied the possibility of using a passive BCI involving the fNIRS combined with an EEG to monitor pilots’ engagement features for cognitive fatigue detection during simulated and real flights [18]. Considering the fNIRS sensor alone, the accuracy score for the cognitive fatigue detection was around 81.5% in the simulation case and 83.2% for the real flight case. However, the individual differences were evident, as individual accuracy scores ranged from around 70–90%. The combination of EEG-fNIRS revealed better results, indicating that additional data sources may be beneficial for the detection of cognitive fatigue.

Zadeh et al. demonstrated that it was possible to detect cognitive fatigue using functional magnetic resonance imaging data with an accuracy of 73% [19].

In the context of cognitive evaluation, the fNIRS Pioneer™, a wearable fNIRS sensor, has been validated and compared to the NINScan device during standard cognitive tasks, both reporting a positive correlation of the results, showing an increase in relative concentration of oxygenated haemoglobin (HbO_2_) with task difficulty and, simultaneously, a decrease in the performance of the user [20].

Trakoolwilaiwan et al. used LABNIRS with 34 channels to monitor motor execution during predefined tasks. In this work, the authors classified three classes: rest, right-hand movement, and left-hand movement. The accuracy scores to classify these classes were around 86–93% using ML algorithms. The lowest results (86%) were obtained using ML trained with custom features extraction, while the highest results (93%), were obtained with automatically extracted features using a convolutional neural network, demonstrating the importance of using appropriate features for the classification of fNIRS signals [21].

However, inter-subject variability has been studied and is reportedly relevant for a proper analysis of fNIRS. On a more general level, age was demonstrated to influence the cerebral haemodynamic [22]. Namely, older people revealed fewer changes in blood oxygenation when considering the ones with a normal pattern of an increase in the concentration of oxygenated haemoglobin and a decrease in the concentration of the deoxygenated haemoglobin during task execution.

On the individual level, Quaresima et al. studied the relative changes of the concentration of the chromophores of haemoglobin relative to the execution of a verbal fluency task, where only half of the population studied, showed significant changes [23]. Zohdi et al. also applied the fNIRS during a verbal fluency task, but added systemic physiological activity (SPA) measurements, namely, heart rate, electrodermal activity, SpO_2_, mean arterial pressure, and P_ET_CO_2_ to study the differences in cerebral haemodynamics [24]. Again, the expected pattern was only visible in half of the population studied.

Holper et al. applied the fNIRS sensors in 11 subjects while performing two tasks: motor imagery and motor execution. On the group level, they demonstrated that it was possible to discriminate between tasks and also between task complexity. Nevertheless, they also verified that the expected pattern of the chromophores was subject dependent, which might contribute to the inter-subject variability expressed in other works [25].

In this work, we propose the development of a physiological-based passive BCI, which applies a two-channel fNIRS sensor to detect cognitive fatigue in the context of e-learning.

## 3. Materials and Methods

### 3.1. Data Acquisition

Cognitive fatigue refers to a process that occurs after executing cognitive functions with high demand over a period of time [26]. Thus, we developed an experimental procedure that involved demanding tasks, namely, a digital lesson in Jupyter Notebook format, Corsi-Block task [27], and a concentration test inspired by the work developed in [28].

While executing the three proposed tasks, the participants’ physiological signals were monitored using two biosignalsplux devices from PLUX Wireless Biosignals, Lisbon, Portugal, with a sampling frequency of 100 Hz and a resolution of 16 bits. Six different sensors were used: EEG and fNIRS positioned around the F7 and F8 of the 10–20 system [29]; ECG monitored Lead I of the Einthoven system, EDA placed on the palm of the non-dominant hand, and the RIP was attached on the upper-abdominal area to measure the respiration rate—the combination of the three allows to infer about the response of the Autonomic Nervous System (ANS) of the human body, namely, the response of the sympathetic and parasympathetic nervous system [30]; ACC on the right side of the head to measure head movement and overall posture changes. All data was recorded using OpenSignals, developed by PLUX Wireless Biosignals, and stored in TXT and HDF5 formats.

Specific to the fNIRS monitoring, the focus of this work, it is important to justify its positioning. Among other brain areas, the dorsolateral prefrontal cortex is involved in the prediction of effort and, thus, the regulation of cognitive fatigue [17]. Thus, we employed the 2-channel fNIRS around the F7 and F8 positions of the standard 10–20 system of the EEG.

Behavioural features, namely, Human–Computer Interaction (HCI) features were continuously extracted using Latent, a Chrome extension to track these features in the context of web-based navigation [31]. Mouse tracking, keyboard strokes, audio, snapshots, and screenshots were recorded and stored in a MongoDB for future analysis.

### 3.2. Sample Description

A data sample of 10 volunteer participants (4 females) aged between 22 and 48 years old (M = 28.2, SD = 7.6) took part in this study. All volunteers were recruited at NOVA School of Science and Technology, fluent in English, right-handed, none reported suffering from psychological disorders, and none reported taking regular medication. Written informed consent was obtained before participating and all Ethical Procedures approved by the Ethics Committee of NOVA University of Lisbon were thoroughly followed.

### 3.3. Data Acquisition Procedure

The experimental protocol is divided into three tasks: a digital lesson, three repetitions of the Corsi-Block task, and two repetitions of a concentration task.

The lesson was about the electrocardiogram (ECG) and was developed in Jupyter Notebook format mainly using HTML, JavaScript, and CSS programming languages. During the task, the participants were required to read theoretical content related to the origins, abnormalities, and overall uses of the ECG signal and, in the end, make a self-evaluation by answering 40 questions about the content they had read. To monitor their reading pattern, all text and images were blurred initially and would only unblur when the mouse would hover over the paragraph/image they were reading/seeing, allowing us to properly track their progress.

**Figure 1:**
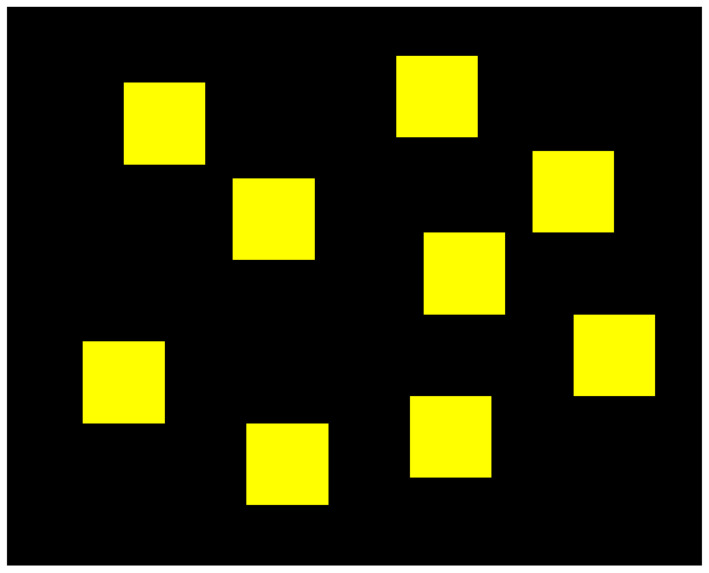
Board of the Corsi-Block task used during the experimental procedure. The yellow blocks would flash in blue by a specific order that the participants would have to memorise and then recall to click the blocks in the same order. The position of the blocks was adapted from [32].

The Corsi-Block task consists of a board containing nine blocks, as illustrated in Figure 1. Firstly, two of those blocks would flash in a specific order and the participants were asked to memorise the order and then click on the same blocks in the same order. If they were successful in recalling the sequence, the level would increase, meaning that three blocks would flash in a different order. This would repeat until the participants reached level nine (because there were only nine blocks) or if they failed two consecutive tries.

**Figure 2:**
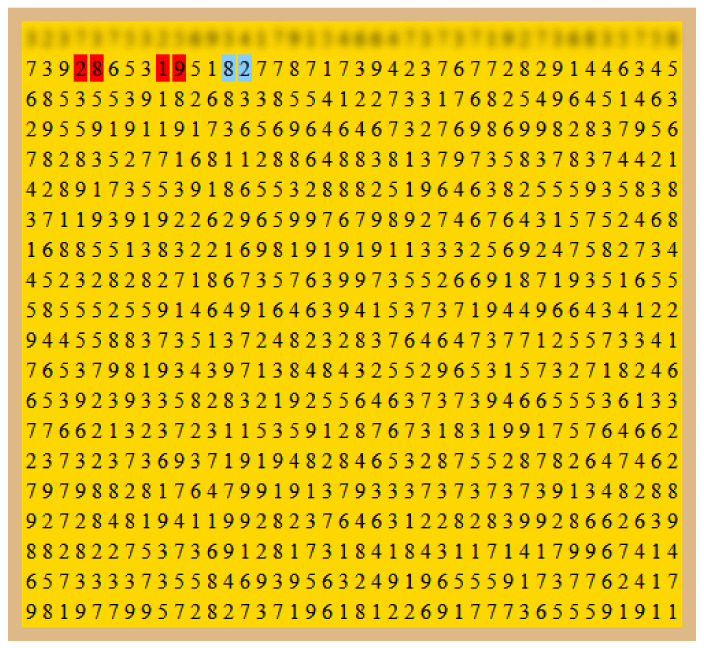
Board of the concentration task. A pair of numbers would turn blue with the mouse hover. If that pair summed to 10, the participant would have to click with the mouse. Whenever they clicked, their positions would be marked in red. Board adapted from [28].

The concentration task consisted of a board of 20 by 40 numbers generated in a pseudo-random fashion. The initial board was generated and, then, a number would be changed with a probability of 20% in order for the sum with the next number to be 10. The participants would be required to go over the board line by line and click on the pairs of consecutive numbers that added to 10. The pairs were marked with blue when they were hovering them and marked in red whenever they were clicked. When they changed line, the previous line was blurred and blocked so that they could not go back. An example of a task is shown in Figure 2 in which the participant was completing the second line of the board (the first line was already blocked).

**Figure 3:**
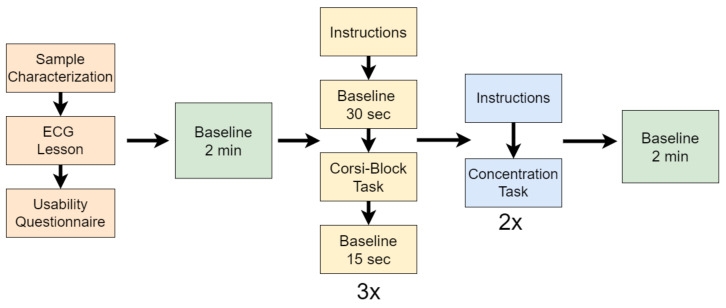
Schematic of the data acquisition protocol. The Corsi-Block task (yellow blocks) is performed 3 times, while the Concentration task (blue blocks) is performed only 2 times.

In addition to the abovementioned tasks, the participants were required to fill out two questionnaires, one at the beginning for sample characterisation and one after the ECG lesson, about their opinion regarding usability (whether the length of the lesson was adequate, whether the language was clear, etc.). Then, the participants solved three repetitions of the Corsi-Block task and, finally, they solved the two repetitions of the concentration task. Before the Corsi-Block task and after the concentration task there were periods of baseline of two min. The first baseline period was labelled as *absence of cognitive fatigue*, while the last baseline period was labelled as *cognitive fatigue*. Between repetitions of the Corsi-Block task, there were periods of baseline of 15 s after the task and of 30 s before the beginning of each repetition of the task, so that the cerebral blood flow would recover after the participants completed the task and after they read the instructions in each repetition, respectively. The experimental procedure is illustrated in Figure 3.

### 3.4. Signal Processing

The acquisition of fNIRS signals requires the presence of two emitting light sources and at least one detector by channel. The emitted radiation transverses the biological tissues of the head, i.e., skin, superficial blood vessels, bone, brain, and blood vessels of the brain, and through scattering effects, reaches the detector with an intensity lower than the emitting intensity mainly due to absorption [33]. Two different emitting lights sources are employed, commonly one in the infrared spectrum and the other in the red spectrum, because the difference in their interaction with the chromophores of haemoglobin is what allows to infer their relative concentration (their interaction with other tissues is usually neglected) [34]. Thus, the measurement is made in terms of variation of intensity between the incident and the detected radiation.

In this case, the first step of signal processing is then to apply the modified Beer–Lambert law, which converts the detected values to variation of concentration of each chromophore, namely, oxygenated haemoglobin (HbO_2_) and deoxygenated haemoglobin (Hb) given specific parameters, namely, emitter-detector distance and extinction coefficient of each wavelength. Equations (1) and (2) show the modified Beer–Lambert law to calculate the relative concentrations of each chromophore [14]. There, *d* represents the emitter-detector distance, which in this case is 2 cm, DPF is the Differential Path Length Factor, $\epsilon_{Hb/HbO_{2}}^{\lambda_{X}}$ represents the extinction coefficient of each wavelength relative to each chromophore of haemoglobin, and *I* represents the light intensity (*b* in the baseline and *t* during task).

(1) $$\Delta HbO_{2}=\frac{\log\frac{I_{b}^{\lambda_{1}}}{I_{t}^{\lambda_{1}}}\epsilon_{Hb}^{\lambda_{2}}-\log\frac{I_{b}^{\lambda_{2}}}{I_{t}^{\lambda_{2}}}\epsilon_{Hb}^{\lambda_{1}}}{d\cdot DPF[\epsilon_{HbO_{2}}^{\lambda_{1}}\epsilon_{Hb}^{\lambda_{2}}-\epsilon_{HbO_{2}}^{\lambda_{2}}\epsilon_{Hb}^{\lambda_{1}}]}$$

(2) $$\Delta Hb=\frac{\log\frac{I_{b}^{\lambda_{2}}}{I_{t}^{\lambda_{2}}}\epsilon_{HbO_{2}}^{\lambda_{1}}-\log\frac{I_{b}^{\lambda_{1}}}{I_{t}^{\lambda_{1}}}\epsilon_{HbO_{2}}^{\lambda_{2}}}{d\cdot DPF[\epsilon_{HbO_{2}}^{\lambda_{1}}\epsilon_{Hb}^{\lambda_{2}}-\epsilon_{HbO_{2}}^{\lambda_{2}}\epsilon_{Hb}^{\lambda_{1}}]}$$

Since the DPF is the same in both cases, it was removed and, thus, the variation of the concentration was calculated in order to the factor (unit of mM/DPF). Given that the wavelengths of the emitters used in this work were 660 nm ($\lambda_{1}$) and 860 nm ($\lambda_{2}$), the extinction coefficients were used according to the work of Matcher et al. [35]:

$\epsilon_{Hb}^{\lambda_{1}}=3.4408\;{\mathrm{mM}}^{-1}{\mathrm{cm}}^{-1}$
$\epsilon_{HbO_{2}}^{\lambda_{1}}=0.3346\;{\mathrm{mM}}^{-1}{\mathrm{cm}}^{-1}$
$\epsilon_{Hb}^{\lambda_{2}}=0.7977\;{\mathrm{mM}}^{-1}{\mathrm{cm}}^{-1}$
$\epsilon_{HbO_{2}}^{\lambda_{2}}=1.2071\;{\mathrm{mM}}^{-1}{\mathrm{cm}}^{-1}$

Additionally to each chromophore, we calculated the variation of total haemoglobin (Hbt), which is simply the sum of the previously mentioned Hb and HbO_2_. After this conversion, the signals were filtered using a second-order band-pass Butterworth finite impulse filter with cut-off frequencies of 0.01–1 Hz, to remove electrical and physiological noise, keeping the most informative frequency band of the fNIRS signals, according to the recommendations in [36].

The baseline signals were then segmented into 10-second windows with no overlap [37] according to the two considered periods—before starting the tasks and after finishing them. A label was attributed to each window, considering if it belonged to the period before the tasks, in which case it was labelled as *absence of cognitive fatigue*, or if it belonged to the period after all tasks, in which case it was labelled as *cognitive fatigue*. This processing procedure resulted in 22 time windows for each participant (11 windows per baseline period), as the last 10 s window of each baseline period was discarded to assure that there was no presence of any task.

### 3.5. Feature Extraction and Selection

After segmentation and labelling, a set of features were extracted from each of the time windows. These features were a mix of general-purpose features extracted using the Time Series Feature Extraction Library (TSFEL) project [38] and features specifically tailored to represent fNIRS signals. These features are briefly described in Table 1. Statistical, temporal and spectral features are the TSFEL features that are thoroughly described and their mathematical formulation is given in the original paper. Custom features were the features implemented in this work.

Thus, given that the fNIRS had two channels and that each channel had three metrics, i.e., Hb, HBO_2_ and Hbt, we end up with 26 × 6 features or 156 features for each data sample. Given the relation between that number and the number of samples for each participant, feature selection is important to reduce the number of parameter training in the classifiers.

As feature selection method, a Recursive Features Elimination algorithm was used, where in each classification fold, a cross-validation was performed using only the training set to obtain the features that optimise the models’ performance. The algorithm is able to select the best features, thus, the number of selected features varies from fold to fold. The model used in this procedure was similar to the employed classifier. The RFECV method of the scikit-learn Python package was used [40].

### 3.6. Classification Procedure

We used Random Forest (RF) as a classifier model to distinguish between the two states of cognitive fatigue. RF is a supervised learning algorithm that consists of an ensemble of decision trees that are merged to produce more robust results by training each of the decision tree with different sets of data, by employing bagging to randomly sample data from the input dataset. The final decision of the RF consists of aggregating the results of the individual trees, namely, by following a majority-voting system. In this case, we limited the number of decision trees to 10, the number of features at the nodes of the trees were limited to the square root of the number of features, the split criterion was Gini, and the minimum number of samples used in node splitting was 2.

Building on previous work, we designed a user-tuned classification procedure, i.e., we built different models for different participants [39]. For each participant, the models were validated using a stratified 10-fold procedure, in which the whole dataset was divided in ten, keeping the original distribution of classes in both sets.

Models’ performances were assessed using a set of commonly used metrics namely, the accuracy score, precision, recall, and F1-score. The corresponding equations are in Equations (3)–(6), where TP, TN, FP, and FN mean True Positives, True Negatives, False Positives, and False Negatives, respectively, and represent:

True Positive (TP)—samples predicted as *cognitive fatigue* that correspond to that class;

True Negative (TN)—samples predicted as *absence of cognitive fatigue* that correspond to that class;

False Positive (FP)—samples predicted as *cognitive fatigue* that correspond to the class of *absence of cognitive fatigue*;

False Negative (FN)—samples predicted as *absence of cognitive fatigue* that correspond to the class of *cognitive fatigue*.

(3) $$Accuracy=\frac{TP+TN}{TP+TN+FP+FN}$$

(4) $$Precision=\frac{TP}{TP+FP}$$

(5) $$Recall=\frac{TP}{TP+FN}$$

(6) $$F1-Score=\frac{2\cdot (Precision\cdot Recall)}{\left(Precision+Recall\right)}$$

Additionally, the area under the receiver operating characteristic curve (AUC-ROC) and the corresponding confusion matrices are reported.

## 4. Results

The duration of each task, the total duration of the agglomerate of the tasks, and the average time to complete each task is presented in Table 2. Given that the tasks did not have time limits, their duration varied among individuals.

Analogously, given our approach of developing user-tuned classifiers to detect the state of cognitive fatigue, we will report the results of each individual model relative to each participant. Additionally, since our classification periods were of approximately 2 min, or around 120 s, that we segmented into 10 s windows, as mentioned in Section 3.4, each participant ended up with a total of 22 data samples, each containing 152 features. However, after feature selection, each data sample was composed of less features (varied among each cross-validation fold). Since the datasets were balanced, there was no need to apply any type of downsampling or upsampling.

Stratified 10-fold divides each dataset into 10 train and test sets, thus, each test set was composed of one or two data samples. Models’ evaluation was performed in 10 different folds, thus, our metrics represent the final metrics, i.e., the gathered results of all folds to calculate the metrics at the end of the whole procedure.

**Figure 4:**
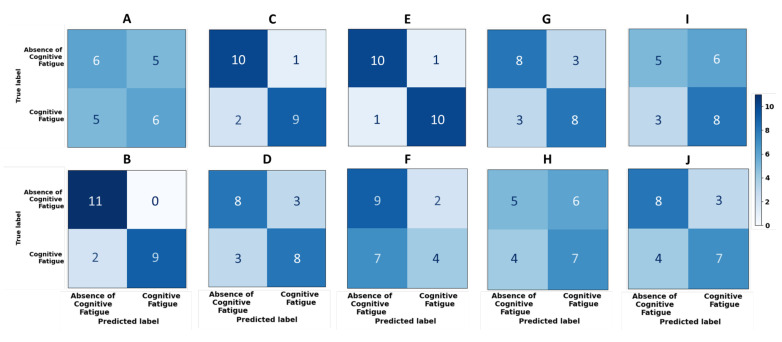
Confusion matrices of each participant, where each letter from **A**–**J** refers to each corresponding participant, respective to the results presented in Table 3.

The classification results for each individual are shown in Table 3 and the respective confusion matrices are illustrated in Figure 4.

## 5. Discussion

The main objective of this work was to study and assess the feasibility of detecting cognitive fatigue using fNIRS wearable sensors. To accomplish that, an experimental protocol was developed to effectively induce cognitive fatigue that involved an ECG lesson, which corresponds to a close-to-real learning session, and the execution of standard cognitive tasks, namely, the Corsi-Block task and a concentration task. fNIRS data was collected during the execution of those tasks, along with data from other physiological signals, in an unobtrusive way. fNIRS data were processed and features were extracted and selected to properly represent the two considered classes. Finally, user-tuned classifiers were trained and validated to automatically detect the state of cognitive fatigue.

According to previous work, cognitive processes are subjective to each individual and, thus, classification models should be developed on the individual level, rather than be developed for a general population [22,23,24,25,39]. Therefore, we built user-tuned models for each participant. The results of classification corroborate the hypothesis that different individuals respond differently to the same demands, since the accuracy results range from 54.55 to 90.91% (mean value 70.91 ± 13.67%). The confusion matrices of Figure 4 show that when the results for the accuracy are higher than 70%, the predicted labels are positively correlated with the true labels and, thus the diagonal of the matrices is more populated. Considering only such cases, the mean accuracy is 82.73 ± 8.33 %, which is in line with other works (e.g., [18]). In cases where the classification is lower than 70%, the expected labels and the predicted classes do not seem to be related.

Moreover, we conclude that the models for cognitive fatigue classification of participants B, C, D, E, G, and J (ages from 23 to 34 (M = 27, SD = 4); 2 females) were successful in distinguishing between *cognitive fatigue* vs. *absence of cognitive fatigue*, while the remaining were unable to effectively distinguish the two cognitive states (see Table 3). In the cases where the accuracy score is close to 50%, namely, participants A, F, H, and I (ages ranging from 22 to 48 (M = 29, SD = 11); two females), it is also possible to note that neither class is preferable because the values of precision, recall, and AUC-ROC are also closer to 50% than the others. On the other hand, in the cases where the models are applicable, precision and recall are balanced meaning that the models are not biased towards either class.

Time is usually regarded as a crucial aspect for the appearance of cognitive fatigue. However, our results demonstrate that it is not the main factor. For example, while participant C took less time to complete the tasks and participant J took the longest time, the classification results did not reflect that difference (see Table 2). Moreover, contrary to the findings of [22], age did not influence the results, since that the groups of participants with accuracy scores higher than 70% and close to 50% did not reveal relevant age differences. Moreover, sex was also not a factor that interfered with the prediction of cognitive fatigue.

Given the disparity of results, it seems clear that, although the employed methodology was very effective for inducing and detecting cognitive fatigue in some cases (e.g., participants B and E), automatic cognitive fatigue assessment using the proposed methodology is not applicable in all cases. We hypothesise that this can be the case due to some aspects: the experimental procedure may not induce sufficient cognitive fatigue in some cases; the extracted and selected features may not be applicable to the cognitive responses for everyone; there may be other factors contributing to cognitive fatigue, e.g., time of day and the regular schedule of work of each individual; how individuals view the tasks (some individuals may try hard to get everything right while others may be less motivated to perform the tasks).

In comparison to other works, our methodology employs fNIRS wearable sensors, which can be easily applied outside the laboratory, which does not happen with the sensor used by Trakoolwilaiwan et al. that uses an fNIRS sensors with 34 channels [21]. Moreover, given that we measure physiological signals over time, the mentioned disadvantages about the subjective and behavioural methods do not apply. However, there is a disadvantage, which is the fact that we assume the state of the participants, i.e., it is assumed that they present cognitive fatigue at the end of the procedure, which may not be the case, as previously discussed.

## 6. Conclusions

In this work, we aimed to develop a methodology to induce cognitive fatigue and to develop ML models able to automatically detect the state of cognitive fatigue using wearable fNIRS sensors with only two channels.

The developed experimental procedure revealed effective and user-tuned models were built to account for individual differences regarding cognitive fatigue. However, it was clear that the methodology was not effective for everyone, i.e., although three models achieved accuracy results higher than 80%, four other models could only accurately classify around 50-60% of the data samples. Thus, to apply the proposed methodology in real-settings, a calibration procedure should be first followed. Then, only if the validated results revealed to be positive, should the resulting models be applied.

In the future, this methodology will be used in real e-learning settings. Since the fNIRS sensors are unobtrusive, they can be used to continuously monitor the cognitive state of e-learning users and advise them about their cognitive fatigue state. Then, users would be able to effectively take breaks to optimise their study sessions.

Finally, the remaining physiological signals and HCI variables will be studied on their applicability on this context, hopefully overcoming the applicability to the participants where fNIRS was not effective.

## Figures and Tables

**Table 1:** Features extracted using the TSFEL Python package. Their respective domain and a short description are also given in the table. Adapted from [39]. Reproduced with permission from Gamboa et al., “Computing, Electrical and Industrial Systems 2021”, Computers; published by MDPI, 2022 [39].

Domain	Feature	Description
Statistical	Maximum	Maximum value of a segment.
	Minimum	Minimum value of a segment.
	Polarity	Maximum of segment divided by the minimum.
	Mean	Mean value of a segment.
	Variance	Variance value of a segment.
	Standard Deviation	Standard deviation value of a segment.
	Kurtosis	Kurtosis of a segment.
	Skewness	Skewness of a segment.
Temporal	Mean of Differences	Mean of the derivate of the segment.
	Total Energy	Total energy of a segment in the form of the sum of the squares of all values divided by the time of the segment.
	Area Under the Curve	Area under the curve of the segment using the trapezoid rule.
	Absolute Energy	Same as total energy, but without accounting for the time of the segment.
	Peak to Peak Distance	Absolute value of the peak to peak amplitude of a segment.
	Entropy	Shannon entropy of a segment.
	Slope of Linear Regression	Slope of a linear regression of a segment.
	Zero Crossing Count	Number of times the signal crosses the zero value.
Spectral	Fundamental Frequency	Frequency of the first prominent peak of the segment’s frequency spectrum.
	Maximum Frequency	Maximum frequency of the segment’s frequency spectrum.
	Power Bandwidth	Width of the frequency band in which 95% of its power is located.
	Spectral Distance	Sum of the difference between the frequency spectrum of the segment to the linear regression of the cumulative frequency spectrum.
	Median Frequency	Median frequency of the segment’s frequency spectrum.
	Spectral Entropy	Spectral entropy of the segment’s frequency spectrum.
Custom Features	Root Mean Square	Root mean square of the fNIRS signal.
	Slope of the Naive Linear Regression	Value of the last data point minus the value of the first data point.
	Maximum Variation	Maximum differential of the signal where each point represents the mean of the following second.
	Minimum Variation	Maximum differential of the signal where each point represents the mean of the preceding second.

**Table 2:** Time each participant spent on each individual task.

Participant	ECG Lesson	Corsi-Block	Concentration	Total
A	16 m 05 s	7 m 12 s	26 m 32 s	49 m 50 s
B	23 m 42 s	5 m 02 s	19 m 51 s	48 m 36 s
C	10 m 12 s	5 m 49 s	17 m 25 s	33 m 26 s
D	11 m 55 s	6 m 18 s	20 m 24 s	38 m 38 s
E	19 m 56 s	6 m 12 s	19 m 27 s	45 m 35 s
F	18 m 48 s	4 m 38 s	30 m 24 s	53 m 50 s
G	17 m 02 s	4 m 47 s	20 m 00 s	41 m 49 s
H	18 m 34 s	4 m 41 s	23 m 18 s	46 m 33 s
I	20 m 05 s	5 m 58 s	22 m 16 s	48 m 19 s
J	20 m 40 s	4 m 47 s	28 m 54 s	54 m 21 s
Average	17 m 42 s	5 m 32 s	22 m 51 s	46 m 05 s

**Table 3:** Classification results for each individual for the task of detecting cognitive fatigue. All results are in percentage.

Participant	Accuracy	Precision	Recall	F1-Score	AUC-ROC
A	54.55	54.55	54.55	54.55	63.64
B	90.91	100.00	81.82	90.00	95.87
C	86.36	90.00	81.82	85.71	86.78
D	72.73	72.73	72.73	72.73	77.69
E	90.91	90.91	90.91	90.91	93.80
F	59.09	66.67	36.36	47.06	44.63
G	72.73	72.73	72.73	72.73	85.12
H	54.55	53.85	63.64	58.33	48.76
I	59.09	57.14	72.73	64.00	59.92
J	68.18	70.00	63.64	66.67	69.83
Average	70.91±13.67	72.86±15.32	69.09±14.77	70.27±14.30	72.50±17.26

## Data Availability

At this moment, data are only available from the corresponding author upon request as it is being prepared to be published.
